# Preliminary experience in laparoscopic resection of hepatic hydatidectocyst with the Da Vinci Surgical System (DVSS): a case report

**DOI:** 10.1186/s12893-017-0294-y

**Published:** 2017-09-11

**Authors:** Haibo Zou, Lanyun Luo, Hua Xue, Guan Wang, Xiankui Wang, Le Luo, Yutong Yao, Guangming Xiang, Xiaolun Huang

**Affiliations:** Department of Hepatobiliary Surgery, Sichuan Academy of Medical Sciences and Sichuan Provincial People’s Hospital, #32, Section 2, West 1st Ring Road, Chengdu, 610072 China

**Keywords:** Hepatic hydatidectocyst, DVSS, Laparoscopic operation

## Abstract

**Background:**

At present, Da Vinci robotic assisted hepatectomy has been routinely carried out in conditional units. But there is no report concerning the use of Da Vinci robots for hepatic hydatid cystectomy and experience on this aspect is seldom mentioned before. This study was to summarize the preliminary experience in laparoscopic resection of hepatic hydatidectocyst with the Da Vinci Surgical System (DVSS).

**Case presentation:**

A 29-year-old female diagnosed as hepatic hydatid in the right anterior lobe of liver was treated with laparoscopic resection by the DVSS under general anesthesia. Appropriate disposal of tumor cell in vascular system and disinfection of surgical field with hypertonic saline were conducted. The hepatic hydatidectocyst was resected completely with an operation time of 130 min, an intraoperative blood loss of 200 ml and a length of hospital stay for five days. The vital signs of patient were stable and no cyst fluid allergy occurred after operation.

**Conclusions:**

Our result showed that laparoscopic resection of hepatic hydatidectocyst by using the DVSS is safe and feasible on the basis of hospitals have rich experience in treatment of cystic echinococcosisliver, resection with DVSS and laparoscopic excision.

## Background

As a common parasitic disease, cystic echinococcosis (echinococcosisgranulosa) is distributed worldwide [1]. In many developing countries, such as Peru, Argentina and western China, cystic echinococcosis is considered an epidemic [2]. For patients with cystic echinococcosis, surgical resection has been the gold standard for this disease. In recent years, alternative methods have facilitated the treatment of specific patients and now become first-line treatment options. Conservative treatment, percutaneous therapy, surgical resection and chemotherapy are the most common treatment strategies. [3]. With the advancement and improvement of laparoscopic technique, laparoscopic hepatectomy is becoming more and more mature in recent years [4, 5], which provides a technical basis for minimally invasive surgery for hepatic hydatidosis [6]. Minimally invasive surgical treatment of hepatic hydatid disease is considered to be safe [7]. However, for the cases with poor exposure and complex operation, laparoscopic surgery has its inherent shortcomings which limit its application.

Over the last decade, robotic surgery has played an increasingly important role in gynecology [8, 9]. The superiority of robot assisted laparoscopic surgery have been reported in many papers. At present, robotic surgery is widely used in a variety of surgical procedures, such as cholecystectomy, esophagectomy, thymectomy, et al. [10]. The advantages of robotic surgery mainly include three dimensional (3D) vision, reduced operator fatigue, more dexterous and penetration of the surgeon’s natural tremor. [11, 12]. The robot-aided thymectomy was firstly conducted by Yoskino et al. for the treatment of thymoma. Their experience suggested that a surgical robot has a great potential for facilitating precise surgery in a narrow area [13]. During surgery, a doctor operates the DVSS arms inserted through the surgical ports used in conventional Video-assisted Thoracoscopic Surgery. In addition, the endoscope of DVSS could provide a 3D high-resolution binocular view of the surgical field, which is far larger than that of video-assisted thoracoscopic surgery, thus the surgical convenience and safety is improved. [14]. The operability around anatomic structures was improved as the Endo-Wrist system can articulate and rotate 360 degrees [15]. Furthermore, the physiological vibrations of the surgeons’ hands can be eliminated via the Endo-Wrist system [14].

Based on the mature technology of laparoscopic resection of hepatic cystic echinococcosis, the world’s first Da Vinci robot assisted resection of hepatic cystic echinococcosis was successfully implemented in 11th March, 2016. Now the preliminary treatment experience are reported as follows.

## Case presentation

**Fig. 1:**
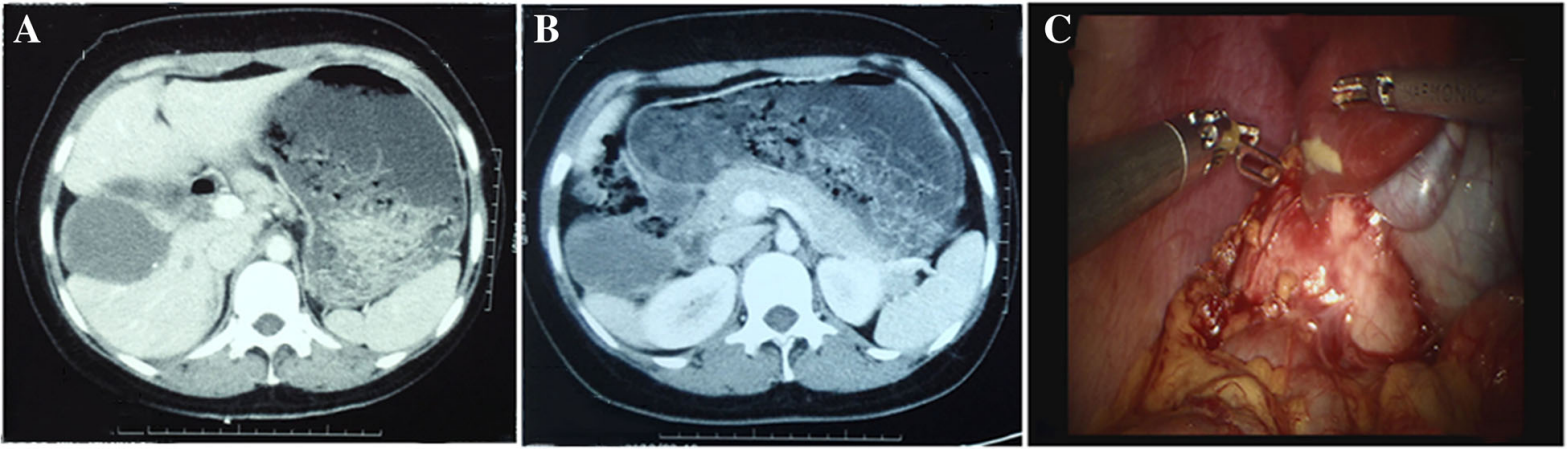
**a** CT showedabnormal morphology of the right liver, spins to the right; **b** CT showed a hepatic hydatid cyst located in the anterior segment of the right lobe of the liver; **c** Intraoperative visual field showed hydatid cysts adjacent to the gallbladder and liver spins to the right

A 29-year-old woman of Han nationality with a history of chronic hepatitis B presented more-than-one-year persistent right epigastric pain. She was a long-term resident of the pastoral area of Aba in Sichuan Province and raised dogs at home. The BMI assessed at admission was 23.9 kg. m^2^, the nutritional risk score was 1, the ability of daily living (ADL) grade was I and ECOG score was 0. Physical examination showed normal vital signs; skin and sclera without yellow dye; no abnormal cardiopulmonary function and palpable superficial lymph nodes; liver and spleen were impalpable. Auxiliary examination showed HBsAg(+), HBeAg(+), HBcAb(+), Pre-S1Ag(+);HBV-DNA:5.7E + 04 IU/ml;ALT: 118 U/L, AST: 92 U/L,normal PT, ALB, TBIL, liver function: Child A; ICG R15:5.4% (DDG-3300 K, Nihon Kohden); normal serum AFP (chemiluminescence analysis); liver ultrasound imaging: hepatic cystic echinococcosis; contrast-enhanced CT: no hydatid lesion was found in head and lungs; an about 6 cm cystic mass with a little marginal calcification, which down into the gap of liver and kidney, was found on the anterior lobe of the right liver (Fig 1 and 1b). The cystic fluid was slightly turbid, and the cystic mass was diagnosed as hepatic cystic echinococcosis.

Half an hour before surgery, a dose of antibiotics (ceftazidime, 1.0 g) was used to prevent infection. The patient was treated with 100 mg hydrocortisone and rapid fluid infusion of 2000 ml before operation, the CVP was required to fall below 12 cm H_2_O during operation. After general anesthesia and tracheal intubation, the patient was placed in a 15° reverse Trendelenburg position (head-high, feet-low). The pneumoperitoneum pressure was maintained at 15 mmHg.

**Fig. 2:**
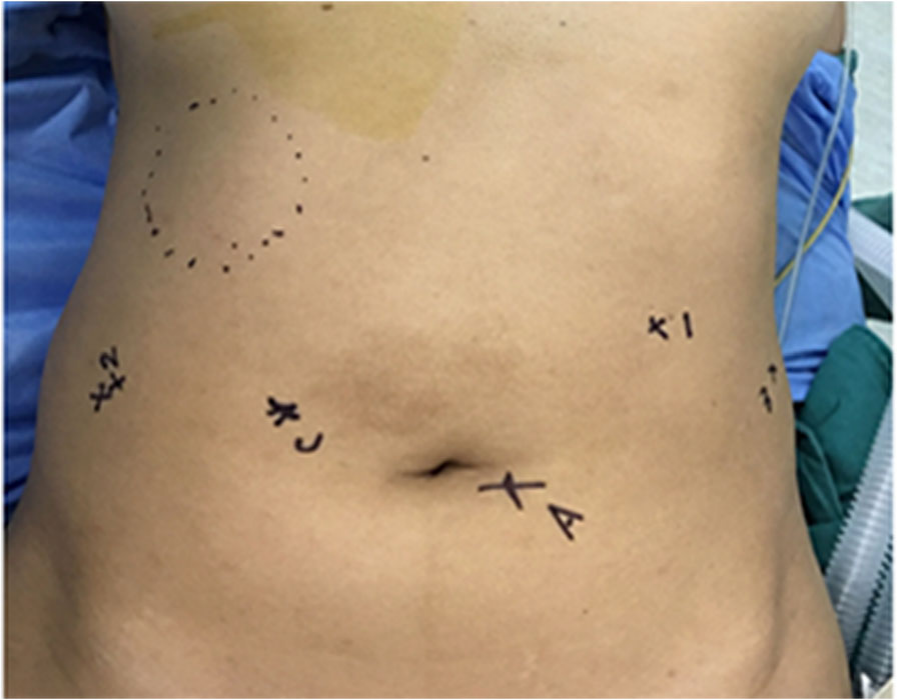
Abdominal wall incisions

The distribution of Troca was shown in Fig. 2 and Da Vinci robot assisted resection was performed as follows: The right side of the umbilicus was selected as observation hole and the left side of the umbilicus was selected as auxiliary hole; No. 1 arm located below the left costal margin in the mid-clavicular line; No. 2 arm located 3 cm above the umbilical level on the right anterior axillary line; No. 3 arm located 3 cm above the umbilical level on the left anterior axillary line,. A straight needle with thread was punctured into the round ligament. After crossing the round ligament in the abdominal cavity, the thread was pierced from the opposite side. Lifting the thread to fully expose the surgical site. Then the liver and abdominal cavity was examined after successful puncture.

**Fig. 3:**
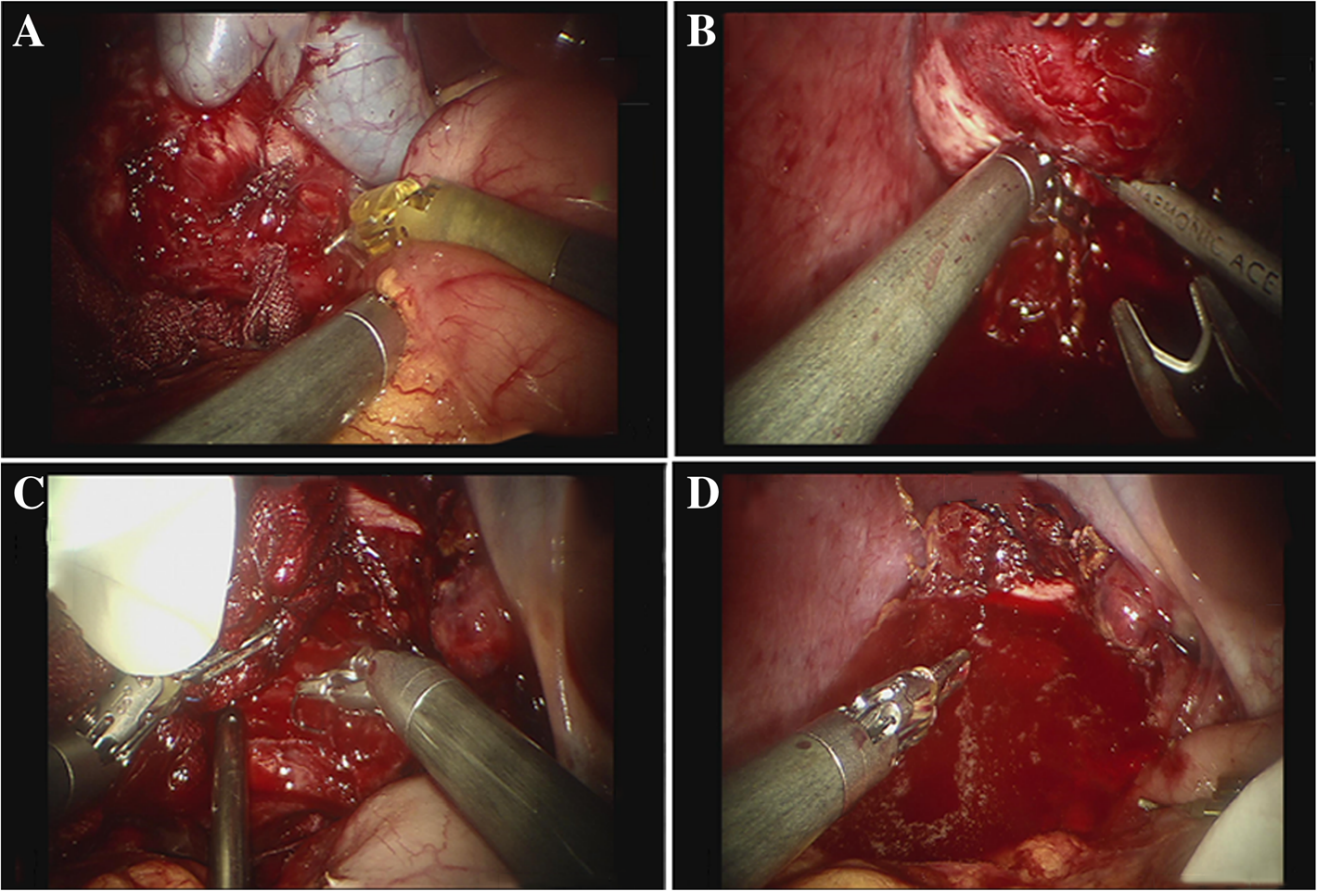
**a** The separation of the omentum and the colon wrapped cyst; **b** Clamping large vessels and bile ducts; **c** Suture the bleeding point; **d** The operative field was soakedwith hypertonic saline

The adhesion between the cyst and omentummajus, colon and stomach was severed with the ultrasound knife (Fig 3a). Free the lower edge of the cyst with the ultrasound knife. The operation should be careful, especially to prevent the rupture of colon. To find the space between fibrous capsule and hepatic tissue, the liver capsule and part of the liver parenchyma was dissected with an ultrasound knife along the junction of hydatid cyst and normal liver tissue. Then the space was deepened gradually via a series of measures such as pushing, squeezing, sub and hook. The blood oozing and crushing tissues were scraped and sucked out by aspirator to make the operation field clearer. When encountered a pipe structure larger than 3 mm, the titanium clip was used for clamping (Fig 3b). During the propulsion process, the cyst was pressed from inside to outside with an aspirator by the assistant to maintain a certain tension and good vision of the space. Promote in the same plane from top to bottom, from front to back, until the specimen was completely free. No visible spill of cystic contents was observed during the operation. Then the outer capsule of the hydatid cyst was completely removed. Biliary ducts were seen during the operation, but no major biliary duct was observed. A complete removal of the external capsule was performed during our operation, so there was no residual cystic cavity, and only the interface between the liver and the cyst were left after resection. So omentum was not used to obliterate the cyst cavity.

After resection, the cyst was loaded into the specimen bag and then the contents of the cyst were suck out with a puncture needle. Bleeding points were treated with electrocoagulation and suture (Fig 3c). Electrocoagulation hook (Johnson & Johnson, US) was mainly used for electrocoagulation of wound oozing blood and small bleeding point. No vessel sealing system was used in this study. Larger tubes (such as portal branch, branch of hepatic vein, small bile duct and hepatic artery) were treated with titanium clips and sutures. Before the end of the operation, the dissection plane of the liver parenchyma and surgical field were immersed with hypertonic saline for 10 min to kill potential echinococcus (Fig 3d) and an abdominal drainage tube was placed in the liver sections postoperatively. The drainage fluid was a pale bloody liquid (mainly from small amount of blood oozing from the wound surface). No bile or other liquid was observed. To prevent recurrence and reinfection, oral albendazole (15 mg/kg/d) therapy against parasites was initiated half a month after surgery and lasted for one year.

After operation, the patient was educated with diet, hand hygiene, and proper knowledge for keeping dogs. The volume of blood loss was about 200 ml during operation. The main cause of bleeding was the small nutrient vessels and the hepatic parenchymal hemorrhage when the hydatid cyst was separated. In addition, since the sixth branch of the middle hepatic vein was close to the cyst, a small break may occur during the process of cyst separation, which also lead to small amount of bleeding. Reliable hemostasis was achieved after suture. The surgery time was 130 min. The patient’s vital signs were stable, no cystic allergies appeared. Subhepatic drainage was left for 24 h. On the first day after surgery, only liquid peroral diet was allowed. No complications such as postoperative bile leakage and bleeding were observed. The patient was discharged 5 days later. Cystic echinococcosis was confirmed by postoperative pathology. Excellent result was obtained in the laparoscopic resection of hepatic hydatidectocyst by the Da Vinci Surgical System (DVSS) in our study.

## Discussion and conclusions

At present, Da Vinci robotic assisted hepatectomy has been routinely carried out in conditional units. But there is no report concerning the use of DaVinci robots for hepatic hydatid cystectomy and experience on this aspect was seldom mentioned before. Through this surgical treatment, we summarized that liver hydatid cyst grows slowly and is prone to produce vascular and bile duct pushing, oppression and infringement. For lesions located on the edge of the liver, the dense pipe system outside the capsule fiber layer should be clamped and sutured to reduce the incidence of postoperative bleeding and bile leakage. Due to the technical advantages of 3D magnification vision and multi DOF manipulator of the Da Vinci robot, the operation is more simple, flexible and precise compared with laparoscopic surgery. For the separation, suture, knotting and other complex operations, the advantages of Da Vinci robot surgery is particularly evident. It can reduce the probability of fluid leakage due to cyst rupture, reduce the operation time, improve the operation precision, and reduce the postoperative complications such as bleeding and bile leakage.

Compared with the traditional operation, the advantages of laparoscopic resection of hepatic hydatid cyst of the external capsule are: 1. compared with liver laparotomy for more than 20 cm of incision, it can greatly reduce the length of the incision and minish the abdominal trauma and scar; 2. It can reduce abdominal adhesions [16], and provide better conditions for the reoperation of possible recurrence; 3. In the operation, the magnified view of laparoscopy can be used to deal with finer bile ducts and blood vessels, and reduce the complications such as bleeding and bile leakage. It can not only achieve the effect of radical resection, but also maximize the retention of normal liver tissue; 4. After surgery, the patient can get out of bed earlier and resume the diet earlier, which shorten the hospitalization time [17]. Da Vinci robotic surgery retains the advantages of laparoscopic surgery, and at the same time, more complex surgery can be implemented because of its excellent operation advantages.

With the improvement of laparoscopic technique, there are almost no restricted areas of hepatic lobectomy[18]. Due to the inherent limitations of laparoscopy and the characteristics of hydatid cysts, more stringent surgical indications are often needed for the implementation of laparoscopic liver hydatid cysts. Poor exposure can greatly increase the risk of rupture and bleeding [19]. Because of its technical advantages, the indications of Da Vinci robot assisted surgery can be more extensive than laparoscopic surgery. Robotic surgery has shown a greater advantage over laparoscopic and open surgery during liver surgery in terms of 3D imaging, increased range of motion within the abdominal cavity and fine control of dissection. Therefore, DVSS may play a vital role in the treatment of posterior cysts close to the vena cava and cysts with thickened and calcified walls.

Preoperative imaging was obtained to assess the relationship between the cyst and the major vessels and bile ducts. The difficulty of surgery will be greatly increased if there is large blood vessel or bile duct invasion. For patients with multiple lesions and the lesions cannot be removed by one-time surgery, the merits of Da Vinci surgery may be extremely limited. Therefore, if there are S7, S8 and other poor exposure lesions, open surgery is still the best choice. For lesions that are well exposed but located at different sites, the distance between the operating arms of the Da Vinci robot should be considered. The interference and collision between the operating arms should be avoided, and the position of Troca should be re-selected if necessary. There are four stages in the surgical methods of hepatic hydatid cyst: internal capsule excision, cyst wall resection, subtotal resection and complete removal of external capsule. Because of high recurrence rate and complications, internal capsule excision and cyst wall resection has been eliminated gradually. At present, the removal of the external capsule and the resection of the liver are considered as radical resection method. Removal of external capsule has increasingly widespread due to its advantage of retention of more normal liver tissue on the premise of radical resection.

Hepatococcal cysts have a gap between the fibrous layer and the liver tissue, which is quite important to identify. It should be noted that this gap is often flat staggered, if the surgery is not timely adjusted, increased bleeding or cyst rupture may occur. Hydatid cyst fluid contains allergens that can cause severe allergic reactions [20]. To prevent the occurrence of allergic reactions, patients were treated routinely with hydrocortisone 200 mg before surgery. Attention should be paid to observe the vital signs of patient during surgery and corresponding preparations for rescue should be made in advance. Once the cyst ruptures, timely and appropriate decompression is needed, and then wet dressing or soaking of hypertonic saline should be performed. The treatment of operative field with hypertonic saline is of great significance for the prevention of cyst recurrence and planting metastasis [21].

Most of the patients with hepatic hydatididae come from pastoral areas, the living environment of which is likely to cause re-infection, thus postoperative antiparasitic drugs and health education are needed. Minimally invasive surgery is the main theme of surgery in 21 century [22]. Through the above operation, we concluded that the indications of Da Vinci robot assisted surgery may be more extensive than laparoscopic surgery. For patients with hydatid cysts extrusion and multiple piping systems, which need to carry out suture and ligation and other complex operations, the advantages of Da Vinci robot assisted surgery are particularly evident. In theory, it can also reduce the risk of allergy, implant and metastasis caused by the rupture of cyst. However, further study and results are still needed.

This case report suggested that laparoscopicresection of hepatic hydatidectocyst with the DVSS is safe and feasible on the basis of hospitals have rich experience in treatment of cystic echinococcosisliver, resection with DVSS and laparoscopic excision.

## References

[1] Budke CM, Deplazes P, Torgerson PR (2006). Global socioeconomic impact of cystic echinococcosis. Emerg Infect Dis.

[2] Craig PS, McManus DP, Lightowlers MW, Chabalgoity JA, Garcia HH, Gavidia CM, Gilman RH, Gonzalez AE, Lorca M, Naquira C (2007). Prevention and control of cystic echinococcosis. Lancet Infect Dis.

[3] Agudelo Higuita NI, Brunetti E, McCloskey C (2016). Cystic Echinococcosis. J Clin Microbiol.

[4] Mathoulin-Pelissier S, Chevreau C, Bellera C, Bauvin E, Saves M, Grosclaude P, Albert S, Goddard J, Le Guellec S, Delannes M (2014). Adherence to consensus-based diagnosis and treatment guidelines in adult soft-tissue sarcoma patients: a French prospective population-based study. Ann Oncol.

[5] Thomas M, Thepot S, Galic N, Jouanne-Pin S, Remoue C, Goldringer I (2015). Diversifying mechanisms in the on-farm evolution of crop mixtures. Mol Ecol.

[6] Powell HA, Baldwin DR (2014). Multidisciplinary team management in thoracic oncology: more than just a concept?. Eur Respir J.

[7] Lai CL, Yuen MF (2013). Prevention of hepatitis B virus-related hepatocellular carcinoma with antiviral therapy. Hepatology.

[8] Iavazzo C, Gkegkes ID (2013). The role of uterine manipulators in endometrial cancer recurrence after laparoscopic or robotic procedures. Arch Gynecol Obstet.

[9] Iavazzo C, Gkegkes ID (2016). Robotic retroperitoneal lymph node dissection in gynaecological neoplasms: comparison of extraperitoneal and transperitoneal lymphadenectomy. Arch Gynecol Obstet.

[10] Tomulescu V, Stanciulea O, Balescu I, Vasile S, Tudor S, Gheorghe C, Vasilescu C, Popescu I (2009). First year experience of robotic-assisted laparoscopic surgery with 153 cases in a general surgery department: indications, technique and results. Chirurgia (Bucur).

[11] Krill LS, Bristow RE (2013). Robotic surgery: gynecologic oncology. Cancer J.

[12] Ohuchida K, Hashizume M (2013). Robotic surgery for cancer. Cancer J.

[13] Yoshino I, Hashizume M, Shimada M, Tomikawa M, Tomiyasu M, Suemitsu R, Sugimachi K (2001). Thoracoscopic thymomectomy with the da Vinci computer-enhanced surgical system. J Thorac Cardiovasc Surg.

[14] Kajiwara N, Kakihana M, Kawate N, Ikeda N (2011). Appropriate set-up of the da Vinci Surgical System in relation to the location of anterior and middle mediastinal tumors. Interact Cardiovasc Thorac Surg.

[15] Hashizume M, Konishi K, Tsutsumi N, Yamaguchi S, Shimabukuro R (2002). A new era of robotic surgery assisted by a computer-enhanced surgical system. Surgery.

[16] Huang G, Lau WY, Shen F, Pan ZY, Fu SY, Yang Y, Zhou WP, Wu MC (2014). Preoperative hepatitis B virus DNA level is a risk factor for postoperative liver failure in patients who underwent partial hepatectomy for hepatitis B-related hepatocellular carcinoma. World J Surg.

[17] Branch Association of Hepatology (2011). Chinese Medical Association, Guide to prevention and treatment of chronic hepatitis (The 2010 edition). Chinese Journal of Internal Medicine.

[18] Lau WY, Lai EC (2008). Hepatocellular carcinoma: current management and recent advances. Hepatobiliary Pancreat Dis Int.

[19] Lau WY, Leung TW, Lai BS, Liew CT, Ho SK, Yu SC, Tang AM (2001). Preoperative systemic chemoimmunotherapy and sequential resection for unresectable hepatocellular carcinoma. Ann Surg.

[20] Lau WY, Lai EC (2007). Salvage surgery following downstaging of unresectable hepatocellular carcinoma--a strategy to increase resectability. Ann Surg Oncol.

[21] Choi JW, Park JY, Ahn SH, Yoon KT, Ko HK, Lee DY, Lee JT, Kim KS, Choi JS, Han KH (2009). Efficacy and safety of transarterial chemoembolization in recurrent hepatocellular carcinoma after curative surgical resection. Am J Clin Oncol.

[22] Morimoto M, Numata K, Kondou M, Nozaki A, Morita S, Tanaka K (2010). Midterm outcomes in patients with intermediate-sized hepatocellular carcinoma: a randomized controlled trial for determining the efficacy of radiofrequency ablation combined with transcatheter arterial chemoembolization. Cancer.

